# Supplementary material for: Plasma Membrane Blebbing Is Controlled by Subcellular Distribution of Vimentin Intermediate Filaments
Source: Cells. 2024 Jan 4;13(1):105. doi: 10.3390/cells13010105 (PMC10778383; doi:10.3390/cells13010105)
Supplement: Supplementary file 1 [file cells-13-00105-s001.zip › Supplementary information.pdf]

Supplementary Information

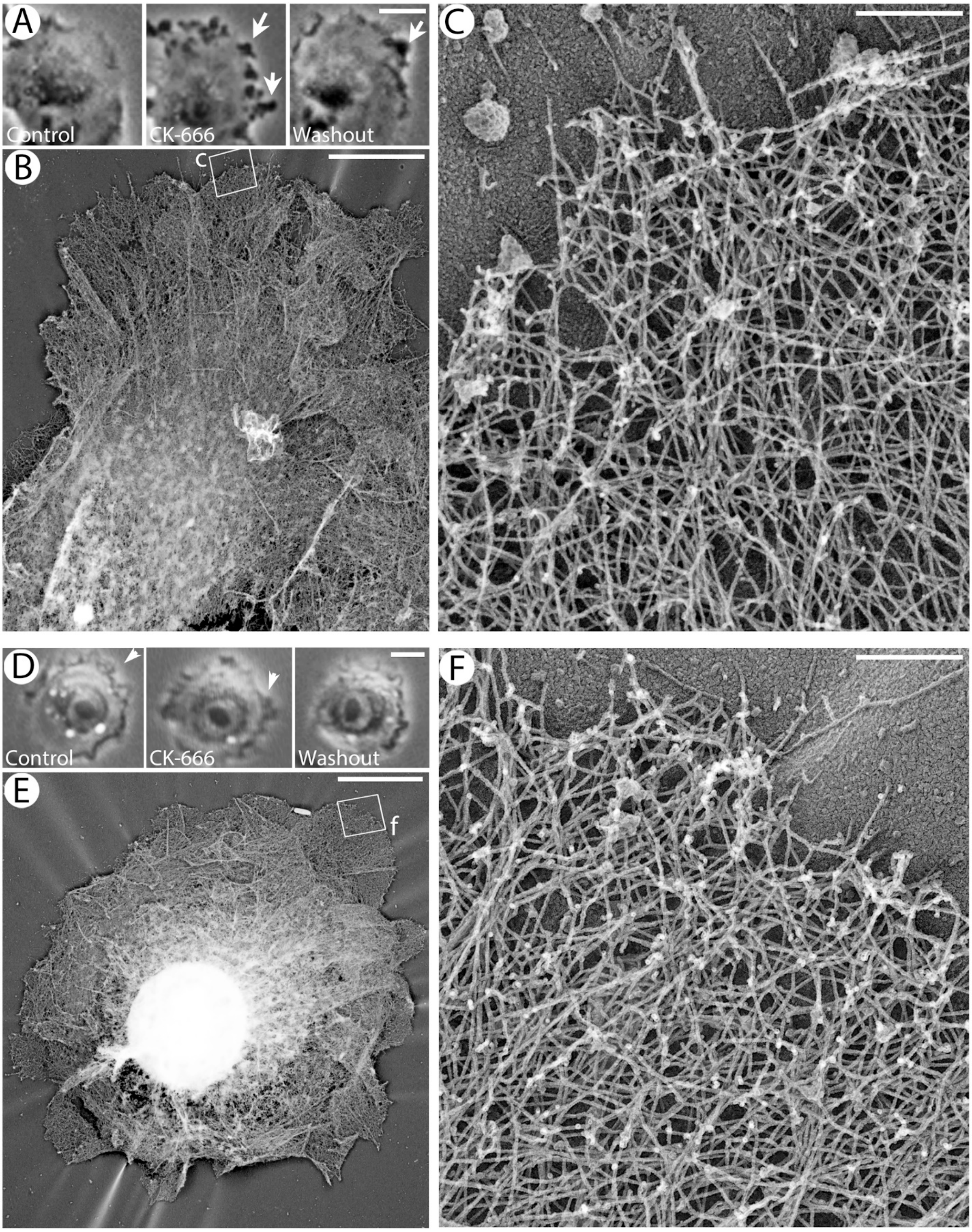

Figure S1. Correlative phase-contrast microscopy and PREM of cells treated with 200 μM CK-666 for 1 h and then allowed to restore lamellipodia after drug washout.

(A-C) Example of a cell that switched to blebbing under CK-666 treatment. (D-F) Example of a cell that did not switch to blebbing under CK-666 treatment. (A, D) Phase contrast images taken before CK-666 application (left), under CK-666 treatment (middle) and after CK-666 washout (right). (B, E) Correlative PREM of the cells shown in A and D, respectively, after detergent extraction, but without gelsolin treatment. (C, F) Enlarged boxed regions from B and E, respectively. Fibrillar structures correspond to actin filaments. Scale bars, 5 μm (A, B, D, E) and 500 nm (C, F).

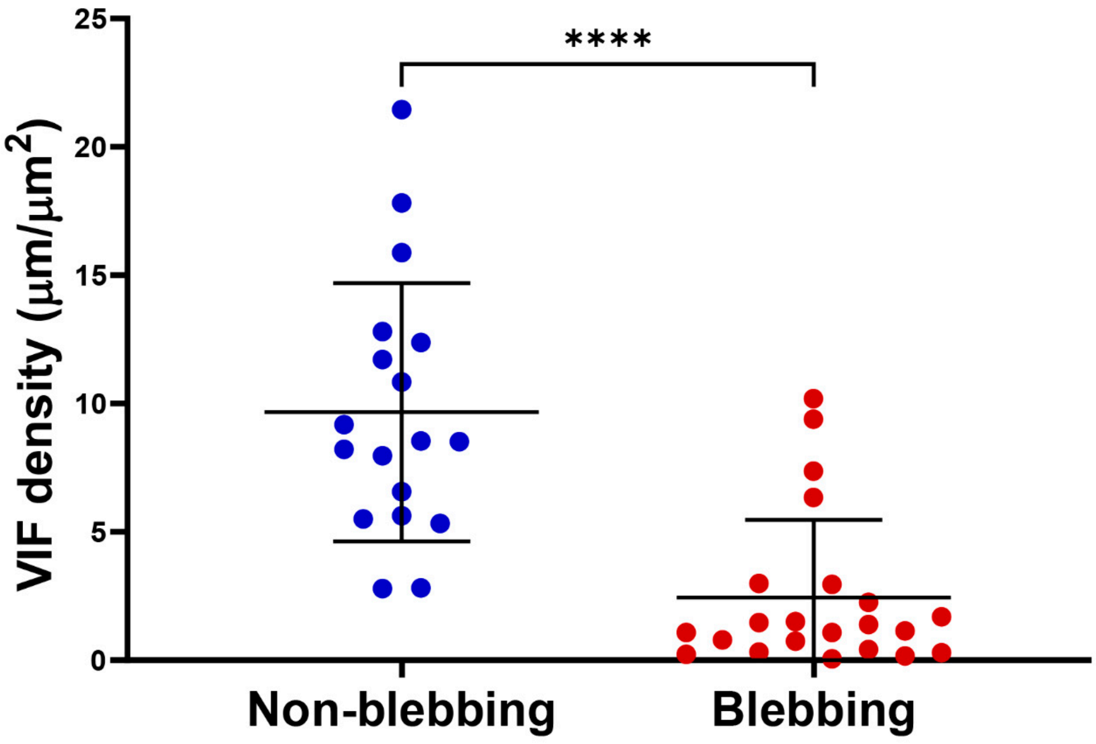

Figure S2. Quantification of VIF density in PREM samples. Each data point indicates area-normalized total VIF length within an individual cell region located adjacent to the leading edge; n=18 regions from 11 cells with total area of 134.36 μm^2 for non-blebbing cells and 22 regions from 6 cells with total area of 183.21 μm^2 for blebbing cells; ****, $p<0.0001$, Mann-Whitney test.

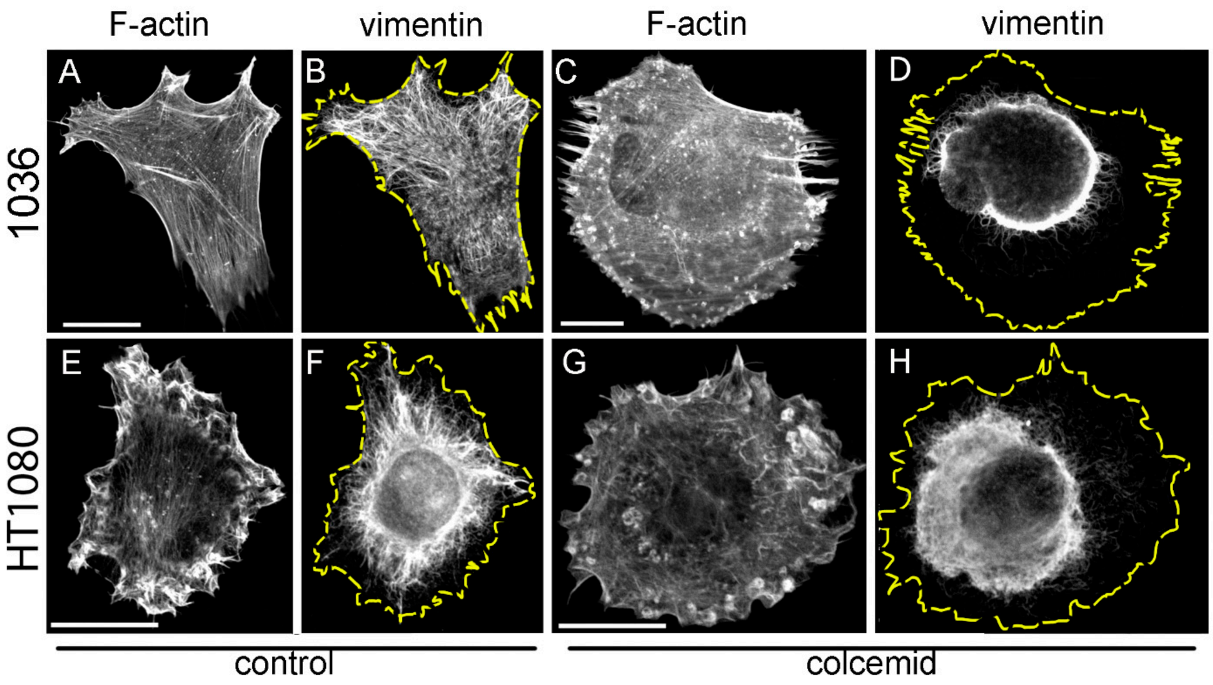

Figure S3. Fluorescence staining of 1036 cells (A-D) and HT1080 (E-H) with phalloidin (F-actin) and vimentin antibody in the presence or absence of 0.2 μM colcemid. Colcemid treatment for 1 h induced a collapse of VIFs. Cell contours in vimentin-stained images (B, D, F, H) are outlined with yellow.

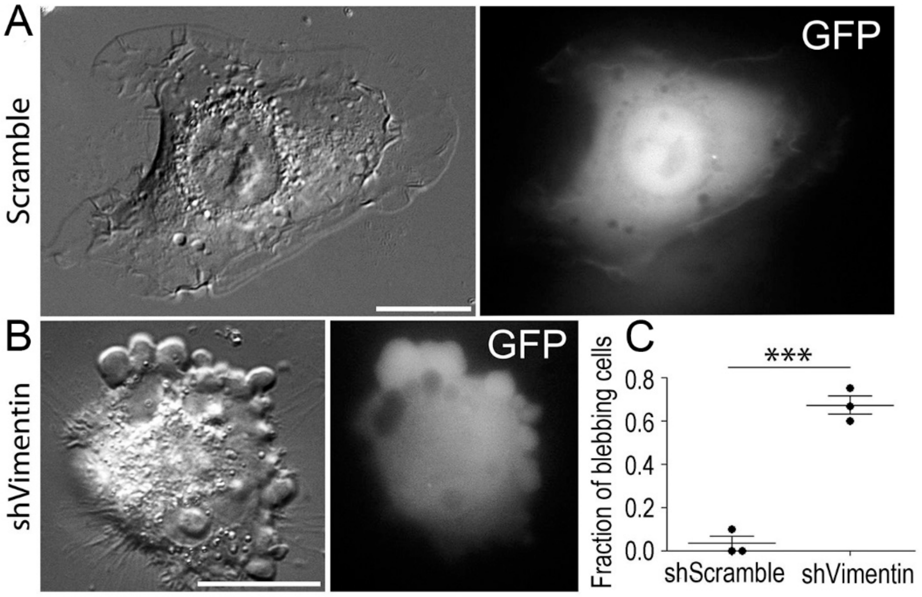

Figure S4. Transient transfection of HT1080 cells with scrambled (A) or vimentin-targeting (B) shRNAs together with EGFP-expressing plasmid. Blebbing is induced in cells transfected with vimentin but not scrambled shRNA even in the absence of CK-666. EGFP panels confirm successful transfection with shRNAs. (C) Fractions of

blebbing cells expressing indicated shRNAs. Data points show individual experiments; lines indicate mean ± SD; n=19, *** p=0.0003, unpaired t-test.

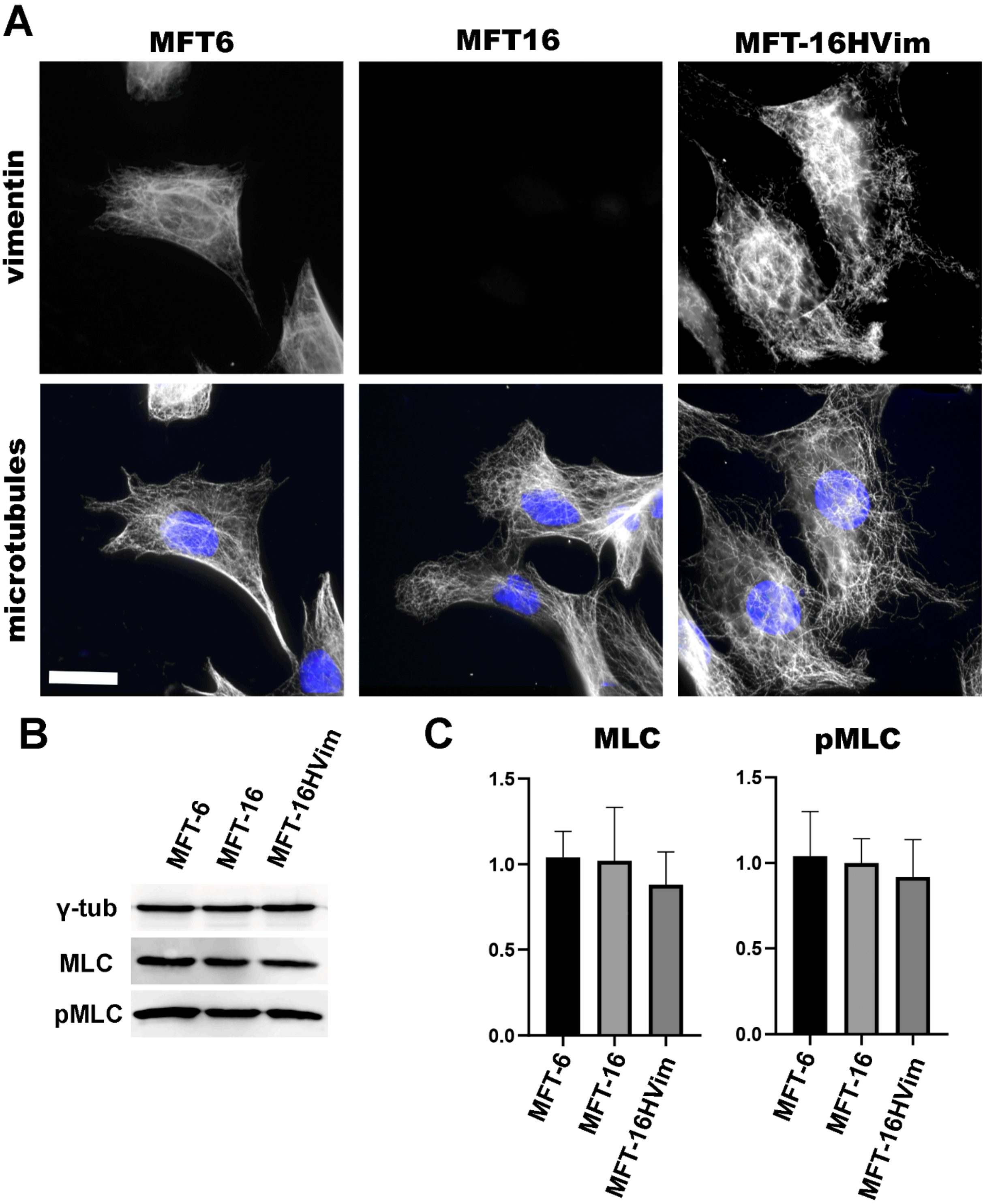

Figure S5. Microtubule distribution and expression levels of MLC and pMLC in MFT-6, MFT-16, and MFT-16HVim cells.

(A) Immunofluorescence staining of VIFs (top) and microtubules (bottom). Scale bar 20 µm. (B, C) Analysis of total MLC and active (phosphorylated) pMLC expression in MFT-6, MFT-16, and MFT-16HVim cells. (B) Western blot of MLC and pMLC. Gamma-tubulin stained by GTU-88 serves as loading control. (C) Quantification of Western blots by densitometry. Mean ± SEM from 5 independent experiments.

Video S1. Treatment of HT1080 cells with 0.2 µM colcemid induces blebbing even without CK-666 treatment. DIC live-cell imaging before and after colcemid treatment. Process is reversible after colcemid washout. Scale bar, 10 µm. Time in min:sec.

Video S2. Treatment with 0.2 µM colcemid induces blebbing in 1036 cells. DIC live-cell imaging before and after colcemid treatment. Scale bar, 10 µm. Time in min:sec.

Macros for image analysis for quantification of center-to-periphery distribution of the mean immunofluorescence intensity of vimentin in cells.

Analysis was done using ImageJ 1.51h version, 64-bit, with Java 1.8.0_66

1. Macro to make mask

```
/// Macro to make Mask

dir=getDirectory("Choose folder with images to analyse");

Extension="_ch01.tif";

dirMask=dir+"Mask"+File.separator();

File.makeDirectory(dirMask);

list=getFileList(dir);


for(file=0;file<lengthOf(list);file++) {

        if(endsWith(list[file],Extension)){
                open(dir+list[file]);
```

```
		shortName=substring(list[file],0,lengthOf(list[file])-lengthOf(Extension));
		run("Duplicate...", "title=temp");
		run("Mean...", "radius=4");
		run("Auto Threshold", "method=Triangle white");
		run("Analyze Particles...", "size=5000-Infinity pixel exclude show=Masks clear");
		run("Maximum...", "radius=10");
		run("Minimum...", "radius=10");
		run("Grays");
		run("Fill Holes");
		saveAs("Tiff",dirMask+shortName+"_Mask.tif");
		run("Close All");
	}
}
```

2. Macro to analyze

```
/// Macrotoanalyze

dir=getDirectory("Choosefolderwith images to analyse");

Extension="_ch00.tif";

dirMask=dir+"Mask"+File.separator();

nbErosionStep=10;

list=getFileList(dir);


run("Set Measurements...", "area mean min centroid center integratedmedianstackredirect=None
decimal=3");

for(file=0;file<lengthOf(list);file++) {
	if(endsWith(list[file],Extension)){
		roiManager("Reset");
		run("ClearResults");
		open(dir+list[file]);
		shortName=substring(list[file],0,lengthOf(list[file])-lengthOf(Extension));
		open(dirMask+shortName+"_Mask.tif");
		run("Duplicate...", "title=distMap");
		run("Distance Map");
		run("Select All");
		run("Measure");
		MaxDist=getResult("Max",0);
		run("ClearResults");

		selectWindow(shortName+"_Mask.tif");
		run("Invert");
		run("CreateSelection");
		roiManager("Add");
		step=round(MaxDist/nbErosionStep);
		for(i=0;i<nbErosionStep;i++) {
			run("Enlarge...", "enlarge=-"+step+" pixel");
```

```
                    roiManager("Add");
                }

                for(i=0;i<nbErosionStep;i++) {
                    selectWindow(list[file]);
                    roiManager("Select",i);
                    run("Measure");
                }

                MeanInt=newArray(nbErosionStep);
                MeanIntNorm=newArray(nbErosionStep);
                for(i=0;i<(nbErosionStep-1);i++) {
                    MeanInt[i]=(getResult("IntDen",i)-
getResult("IntDen",i+1))/(getResult("Area",i)-getResult("Area",i+1));
                }
                MeanInt[9]=getResult("Mean",9);

                Array.getStatistics(MeanInt, minMeanInt, maxMeanInt, meanMeanInt,
stdDevMeanInt);
                sum=meanMeanInt*nbErosionStep;
                for(i=0;i<(nbErosionStep);i++) {
                    MeanIntNorm[i]=MeanInt[i]/sum;
                }

                Array.print(MeanIntNorm);

                run("Close All");
        }
}
```
